# Pathogen-specific kinetics of oxidative burst in camel leukocytes: Influence of serum opsonization on reactive oxygen species production

**DOI:** 10.14202/vetworld.2025.2252-2263

**Published:** 2025-08-09

**Authors:** Salma Al Adwani, Nardin Al Kindi, Abeer Al Hamrashdi, Samir Al Bulushi, Salim M Al Hajri, Jamal Hussen, Waleed Al Marzooqi, Yasmin El Tahir

**Affiliations:** 1 Department of Animal and Veterinary Sciences, Sultan Qaboos University, Muscat, Oman; 2 Laboratories and Animal Research Center, Directorate General of Veterinary Services, Royal Court Affairs, Muscat, Sultanate of Oman; 3 Department of Microbiology, College of Veterinary Medicine, King Faisal University, Al-Ahsa, Saudi Arabia

**Keywords:** *Camelus dromedaries*, luminol chemiluminescence, microbial pathogens, neutrophils, oxidative burst, reactive oxygen species, serum opsonization

## Abstract

**Background and Aim:**

Dromedary camels exhibit unique immune adaptations that enable survival in harsh environments with high microbial exposure. However, the cellular mechanisms underpinning their innate immune responses, particularly oxidative respiratory bursts, remain underexplored. This study aimed to investigate the kinetics of reactive oxygen species (ROS) production in camel leukocytes in response to selected bacterial and fungal pathogens and to assess the effect of serum opsonization on ROS generation.

**Materials and Methods:**

Whole blood from six clinically healthy female dromedary camels was stimulated with opsonized and non-opsonized *Staphylococcus aureus*, *Pseudomonas aeruginosa*, *Escherichia coli*, *Klebsiella pneumoniae* (three strains), and *Candida albicans*. Luminol-enhanced chemiluminescence (CL) assays were used to quantify ROS production over a 3-h period. Colony-forming units were evaluated to confirm microbial viability post-opsonization. Key ROS metrics included area under the curve, peak emission (relative light unit), and time to peak.

**Results:**

Camel neutrophils demonstrated distinct pathogen-specific ROS kinetics. *P. aeruginosa* and *K. pneumoniae* 1705 elicited the highest total ROS on serum opsonization, whereas *S. aureus* and *C. albicans* showed minimal ROS induction. *E. coli* failed to induce a measurable ROS response. Serum opsonization significantly enhanced total ROS production and shortened peak response time for *K. pneumoniae* strains. In contrast, it reduced total ROS output for *S. aureus* and *C. albicans* without significantly affecting their peak kinetics.

**Conclusion:**

This study provides the first comprehensive analysis of microbial-specific ROS production in camel whole blood using a luminol-based CL assay. The findings underscore the variability in camel innate immune responses to different pathogens and highlight the modulatory role of serum opsonization. These insights could inform future strategies in camel immunotherapy, vaccine development, and disease diagnostics.

## INTRODUCTION

The dromedary camel (*Camelus dromedarius*), commonly referred to as the Arabian camel, is a resil-ient species uniquely adapted to arid environments characterized by extreme temperatures and high exp-osure to pathogens. Due to its distinctive anatomical and physiological traits [1], the camel has evolved specialized cellular defences that enable it to withstand thermal and microbial stress [2, 3]. Compared to other domestic species, camels exhibit lower susceptibility to infectious diseases and environmental stressors, a phenomenon attributed to their robust innate and ada-ptive immune responses [3–6].

The innate immune system of camels comprises physical, chemical, and cellular components, with phag-ocytic cells, such as neutrophils, macrophages, and den-dritic cells, playing a central role in early defence [7]. These leukocytes eliminate pathogens through a potent oxidative respiratory burst mechanism. After engulfing pathogens in a phagosome, the structure matures into a phagolysosome, triggering the rapid release of reactive oxygen species (ROS) [8–10].

The oxidative respiratory burst is initiated on microbial recognition, activating the nicotinamide ade-nine dinucleotide phosphate (NADPH) oxidase (Nox2) enzyme complex on the phagosomal membrane. This complex catalyzes the conversion of oxygen to supe-roxide anions, which are subsequently transformed into various ROS metabolites, including hydroxyl radicals (OH^-^), superoxide radicals (O_2_^-^), hydrogen peroxide (H2O2), and hypochlorous acid (HOCl). These ROS may be released either into the phagolysosome or extracellularly at the site of infection. The presence of opsonins, antibodies or complement proteins signif-icantly enhances this response by promoting the recog-nition and uptake of microbes [7, 11].

Oxidative respiratory bursts have been studied across multiple livestock species, including camels, using luminol-based chemiluminescence (CL) assays to quantify ROS production [6]. Luminol amplifies the chemiluminescent signal generated during ROS oxid-ation, offering an indirect yet sensitive method for assessing leukocyte oxidative activity [6, 12, 13]. Such measurements are vital for evaluating the functional capacity of phagocytic cells in microbial killing.

Infections caused by bacterial pathogens pose a significant health challenge in camels and present zoon-otic risks. *Klebsiella pneumoniae* and *Staphyloc-occus aureus* have been identified as primary etiological agents of camel diseases, such as endometritis, pneumonia, and mastitis [14–16]. *Pseudomonas aerug-inosa* is frequently isolated from both healthy and diseased camel respiratory tracts, particularly in young animals, and is notable for its increasing resistance to cephalosporins [17, 18]. In addition, camels have been shown to harbor antimicrobial-resistant (AMR) strains of *Escherichia coli*, including colistin- and carbapene-mase-producing variants with zoonotic potential [19]. *Candida albicans*, a fungal pathogen, is often isolated from the udder and genital tract of camels, with incre-ased virulence in diseased individuals [20].

While substantial research has been devoted to camel immunoglobulins, especially their unique single-chain immunoglobulin G antibodies [21], the cellular aspects of camel innate immunity remain und-erexplored. Most existing immunological studies on camels have focused predominantly on their unique humoral immunity, such as their unconventional single-domain antibodies (variable domain of heavy chain or nanobodies). In contrast, the cellular arm of their immune system, specifically the phagocyte-mediated oxidative burst and its modulation by opsonization, has not been thoroughly investigated. This is particularly significant given the rising incidence of AMR path-ogens in camels, such as *K. pneumoniae*, *S. aureus*, *P. aeruginosa*, and *E. coli*, which pose both animal health and public health risks. Furthermore, although luminol-dependent CL assays have been successfully used to quantify ROS in other species, their application to assess pathogen-specific ROS responses in camel whole blood is unprecedented. Additionally, the differential impact of serum opsonization on the oxidative burst response across various microbial species in camels is poorly understood. These knowledge gaps impede the development of effective diagnostic tools, targeted immunotherapies, and vaccination strategies tailored for camelid species.

The primary aim of this study was to characterize the kinetics of oxidative respiratory burst in dromedary camel (*C. dromedarius*) leukocytes in response to different microbial pathogens. Specifically, the study sought to determine the magnitude and timing of ROS production following stimulation with Gram-positive bacteria (*S. aureus*), Gram-negative bacteria (*K. pneumoniae*, *P. aeruginosa*, and *E. coli*), and the fungal pathogen (*C. albicans*). A secondary objective was to investigate the modulatory effect of serum opsonization on ROS production, using pooled 100% camel serum to mimic physiological opsonization. By utilizing whole blood samples rather than isolated neutrophils, the study aimed to provide a more physiologically relevant assessment of immune function. This work fills a critical gap in camel immunology by offering the first syste-matic evaluation of microbe-specific ROS kinetics using a luminol-enhanced CL assay in camel whole blood. The findings are expected to support future efforts in camel disease diagnosis, vaccine development, and immunotherapeutic interventions.

## MATERIALS AND METHODS

### Ethical approval

Ethical approval was obtained from the Ethics Committee for Animal Use in Research at Sultan Qaboos University (Permit No. SQU/Ec-AUR/2023-2024/5).

### Study period and location

The study was conducted from July 2022 to May 2023 on camels housed in large open enclosures at the Royal Camel Corps (RCC) located in Muscat, Oman.

### Blood collection and sample processing

A total of six clinically healthy adult female dromedary camels (*C. dromedarius*) were randomly selected and used as biological replicates across three independent experiments (two animals per experiment).

Whole blood and matched serum samples were aseptically collected from the jugular vein using 7 mL vacutainer tubes (EUROMED International, Cairo, Egypt). Tubes containing heparin were used for complete blood count (CBC) and CL assays, while plain tubes were used to obtain serum for microbial opsonization. Blood samples were processed immediately following collection.

### CBC

CBC analyses were performed on the same day of blood collection using a VET auto hematology analyzer (PKL PPC 1200H, Paramedical srl, Salerno, Italy) to determine baseline hematological values, including white blood cell (WBC) and neutrophil counts.

### Preparation of microbial stimuli

Clinically relevant pathogens were selected for ROS stimulation assays. These included Gram-positive *S. aureus* American type culture collection (ATCC) 25923 and Gram-negative bacteria *K. pneumoniae* ATCC 1706, 1705, and 700603; *P. aeruginosa* ATCC 27853; and *E. coli* ATCC 25922. The fungal pathogen *C. albicans* ATCC 14053 was also included in the study. All microbial strains were cultured in 5 mL Luria Bertani (LB) broth (Sigma Chemicals Co St. Louis, Missouri, USA) and incubated at 37°C for 1 h with shaking to an optical density (OD_600_) of 0.1.

### Microbial opsonization

To assess the impact of serum opsonization, cultu-red microbes were incubated with 100% pooled camel serum at a 1:10 (v/v) ratio for 10 min at 37°C. These were referred to as opsonized microbes. Non-opso-nized controls consisted of the same strains incubated without serum. All preparations were washed twice with phosphate-buffered saline (PBS, pH 7.4) and resu-spended in PBS for further use.

### Opsonization of zymosan (positive control)

Zymosan A particles (Sigma Chemical Co.) were used as a positive control for inducing respiratory burst. A total of 100 mg of zymosan was suspended in 2 mL PBS and boiled for 1 h with intermittent agitation. The particles were washed 3 times with PBS, resuspended to 50 mg/mL, and incubated with 100% pooled camel serum at a 1:10 ratio for 15 min at 37°C. Opsonized zymosan (OZ) was then washed twice with Roswell Park Memorial Institute (RPMI)-1640 (Sigma Life Science, UK) and resuspended in 4 mL RPMI-1640 for assay use.

### CL assay

**Supplementary Figure 1:**
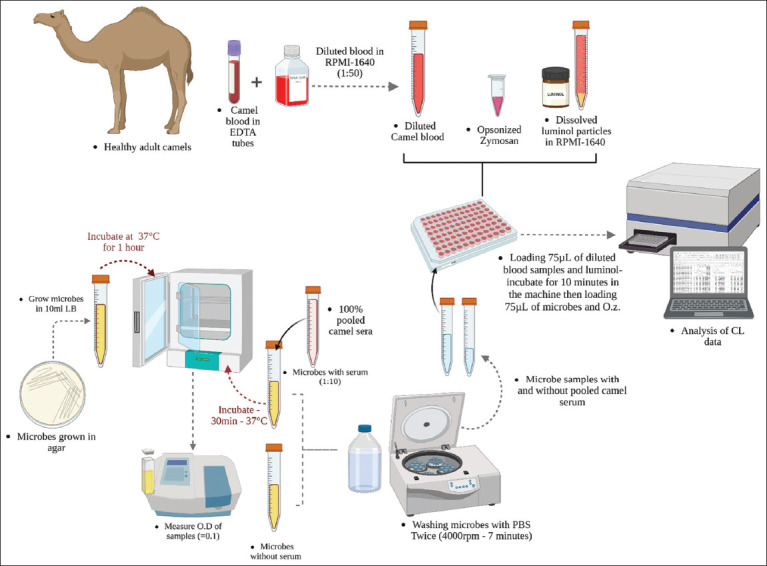
A graphical summary of the luminol-dependent chemiluminescence assay experimental design [https://BioRender.com/6xd08hq].

The oxidative burst was quantified using a luminol-dependent CL assay. Briefly, 75 µL of diluted whole blood (1:50 in RPMI-1640) was added to each well of a 96-well microtiter plate, followed by 75 µL of 20 mmol/L luminol (5-amino-2,3-dihydro-1,4-phthalazinedione). After incubation at 37°C for 10 min, 75 µL of each stimulant (OZ, opsonized microbes, or non-opsonized microbes) was added. Untreated blood served as the negative control. Kinetic readings were recorded every 2 min over a 3-h period using an Ascent Luminometer (Thermo Electron Corp., Finland) with intermittent shaking. Results were expressed in relative light units (RLU). A visual representation of the experimental setup is provided in Supplementary Figure 1.

### Colony forming unit (CFU) assay

To assess whether serum opsonization affected microbial viability, CFU assays were conducted. Microbial strains, both opsonized and non-opsonized, were plated on LB agar (Sigma Chemicals Co.) after treatment and incubated to quantify viable colonies. Comparisons were used to confirm that serum treatment did not significantly impair microbial viability.

### Statistical analysis

All experiments were performed in duplicate. Data were analyzed using GraphPad Prism version 10.2.3 (GraphPad Software, La Jolla, CA, USA) and are reported as mean ± standard error of the mean. The Shapiro–Wilk test was used to assess the data normality. Depending on the distribution, either one-way analysis of variance followed by Tukey’s *post hoc* test or the Kruskal–Wallis test was applied. Unpaired t-tests were used to compare CFU counts, total ROS production, peak CL responses, and time to peak. Statistical significance was set as follows: p < 0.05, p < 0.01, p < 0.001, and p < 0.0001.

## RESULTS

### Hematological profile of camels

**Supplementary Figure 2:**
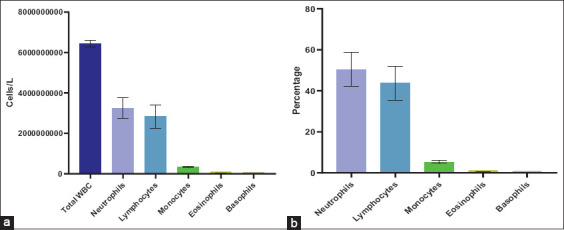
The average complete blood count of three camels’ whole blood was measured using a PKL paramedical product collection 1200 H VET auto hematology analyzer (a) in cells/L and (b) in percentage.

CBC analyses revealed a mean total WBC concentration of 6.44 × 10^9^ cells/L in the sam-pled dromedary camels. Neutrophils represented the pre-dominant leukocyte subset, averaging 3.24 × 10^9^ cells/L (50.49%), followed by lymphocytes at 2.28 × 10^9^ cells/L (43.69%), and monocytes at 0.34 × 10^9^ cells/L (5.31%). Basophils and eosinophils were negligible (Supplementary Figure 2). These hematological para-meters align with published reference ranges for healthy *C. dromedarius* [22]. Based on these results, neutrophils were assumed to be the primary contributors to oxidative burst responses in the subsequent assays.

### Evaluation of microbial viability after serum opsonization

**Figure 1:**
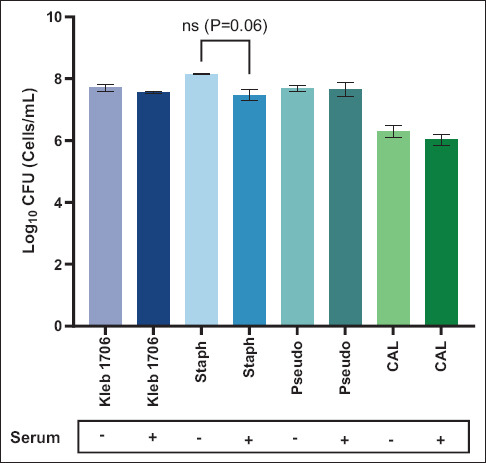
Comparison of microbe colony forming units with and without the addition of 100% camel pooled sera in Log10 cells/mL. Results are presented as average +/− standard error of the mean of three measurements performed on different days and analyzed using the unpaired t-test. Kleb 1706=*Klebsiella pneumoniae* American Type Culture Collection (ATCC) 1706; Staph=*Staphylococcus aureus* ATCC 25923; Pseudo=*Pseudomonas aeruginosa* ATCC 27853; and CAL=*Candida albicans* ATCC 14053.

Before ROS measurement, CFU assays were performed to determine whether serum opsonization affected microbial viability. Microbial suspensions were incubated with 100% pooled camel serum for 30 min, and CFU counts were compared with their non-opsonized counterparts. As shown in Figure 1, no significant differ-ences in CFU counts were observed across all tested strains, indicating that serum treatment did not impair microbial viability and would not confound the oxidative burst measurements.

### CL Assay

#### Kinetics of ROS production

**Figure 2:**
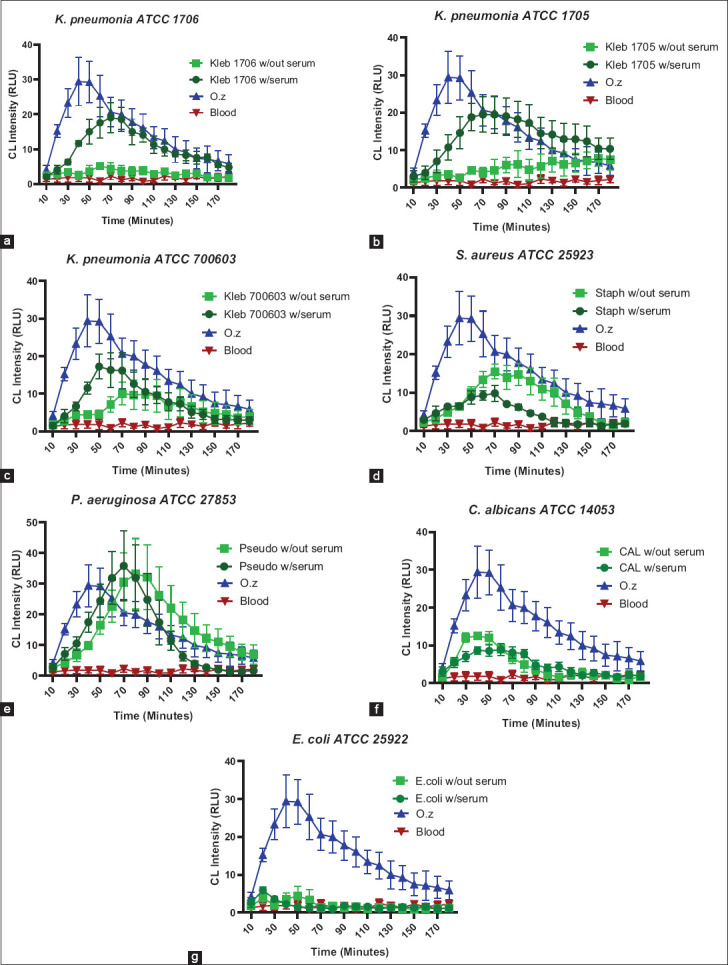
Kinetics of reactive oxygen species (ROS) production in camel’s whole blood against (a) *Klebsiella pneumonia* *1706*, (b) K. pneumonia 1705, (c) *K. pneumonia* 700603, (d) *Staphylococcus aureus* (e) *Pseudomonas aeruginosa*, (f) *Escherichia coli*, and (g) *Candida albicans*, with and without opsonization with 100% camel pooled sera. ROS production was measured using a luminol-enhanced chemiluminescence assay over a 3-hour period. Results are presented as average +/− standard error of the mean of three measurements performed on different days and analyzed by one-way analysis of variance with Tukey’s or Kruskal–Wallis *post hoc* test based on normality test. w/out, without serum; w/serum, with serum; Kleb 1706, 1705, 700603 *Klebsiella pneumoniae* American Type Culture Collection (ATCC) 1706, 1705, 700603; Staph, *Staphylococcus aureus* ATCC 25923; Pseudo, *Pseudomonas aeruginosa* ATCC 27853; and CAL, *Candida albicans* ATCC 14053.

Luminol-enhanced CL was used to monitor real-time ROS generation in camel whole blood following microbial stimulation. OZ was used as the positive control, inducing a robust signal (~30 RLU), whereas untreated blood served as a negative control (<3 RLU) (Figure 2).

*K. pneumoniae* 1706: Serum opsonization signi-ficantly enhanced ROS production compared to non-opsonized samples (p < 0.001). The opso-nized response surpassed the negative control (p < 0.0001) but remained lower than the positive control (p < 0.05) (Figure 2a).
*K. pneumoniae* 1705: While opsonization did not elevate ROS above the positive control, it significantly increased ROS relative to the non-opsonized condition (p < 0.0001) (Figure 2b).
*K. pneumoniae* 700603: Both opsonized and non-opsonized forms showed higher ROS than the negative control (p < 0.05 and p < 0.01, respectively), but their responses were not significantly different from each other (Figure 2c).
*S. aureus*: Both forms induced significant ROS compared to the negative control (p < 0.05 and p < 0.0001), although the opsonized form had significantly lower ROS than the positive control (p < 0.01). Notably, the non-opsonized form sho-wed a stronger response than the opsonized one (Figure 2d).
*P. aeruginosa*: Strong ROS production was induced by both forms, comparable to the positive control (p < 0.01 and p < 0.0001) (Figure 2e).
*C. albicans*: Induced modest but significant ROS production in both opsonized and non-opsonized forms versus the negative control (p < 0.01 and p = 0.05, respectively), with no difference between the two treatments (Figure 2f).
*E. coli*: Failed to elicit any detectable ROS response under either condition (Figure 2g) and was excluded from further ROS analysis.

In summary, opsonization notably enhanced ROS output for *K. pneumoniae* 1706 and 1705 but had minimal or mixed effects on other microbes. Each organism displayed a distinct kinetic ROS signature in response to serum treatment (Figure 2).

#### Total ROS production (area under the curve [AUC])

**Figure 3:**
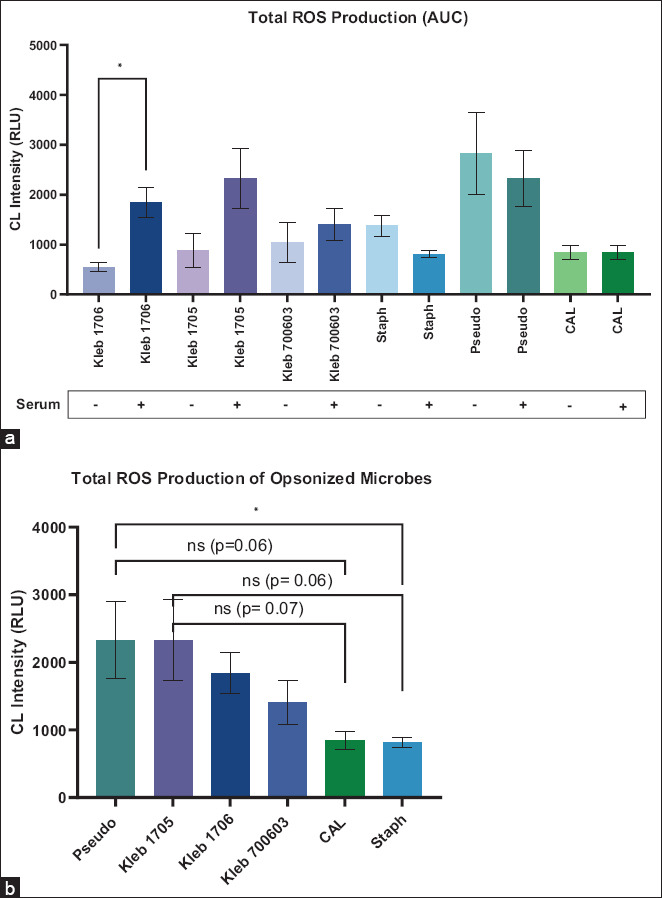
Total reactive oxygen species production was measured as the area under the curve of (a) microbes with and without serum opsonization and (b) opsonized microbes with serum. Results are presented as average +/− standard error of the mean of three measurements performed on different days and analyzed using the unpaired t-test (*p < 0.05). Kleb 1706, 1705, 700603, *Klebsiella pneumoniae* American Type Culture Collection (ATCC) 1706, 1705, 700603; Staph, *Staphylococcus aureus* ATCC 25923; Pseudo, *Pseudomonas aeruginosa* ATCC 27853; and CAL, *Candida albicans* ATCC 14053.

Total ROS output, quantified by calculating the AUC, revealed marked differences between pathogens (Figure 3a). The highest AUCs were observed for opsonized *P. aeruginosa* (2329 RLU) and opsonized *K. pneumoniae* 1705 (2325 RLU). In contrast, opsonized *S. aureus* and opsonized *C. albicans* had the lowest total ROS production (809 and 844 RLU, respectively) (Figure 3b). For *K. pneumoniae* 1706, serum opsonization significantly elevated total ROS compared to its non-opsonized counterpart (p < 0.05). Interestingly, non-opsonized *P. aeruginosa* and non-opsonized *S. aureus* produced more total ROS than their opsonized versions. For *C. albicans*, total ROS production remained unaffected by opsonization. Thus, opsonization selectively enhanced ROS production in specific strains (*K. pneumoniae*), while others were unaffected or even suppressed by it.

#### Peak CL intensity

**Figure 4:**
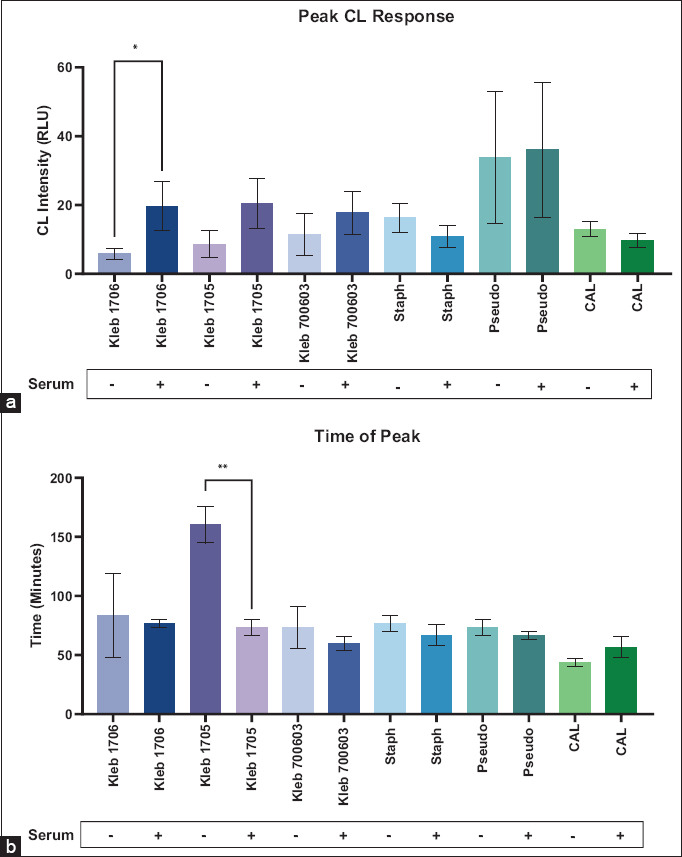
Peak chemiluminescence response measured as relative light units of microbes with and without serum opsonization (a) and peaking time measured in minutes of microbes with and without serum opsonization (b). Results are presented as average +/− standard error of the mean of three measurements performed on different days and analyzed using the unpaired t-test (*p < 0.05, **p < 0.01). Kleb 1706, 1705, 700603, *Klebsiella pneumoniae* American Type Culture Collection (ATCC) 1706, 1705, 700603; Staph, *Staphylococcus aureus* ATCC 25923; Pseudo, *Pseudomonas aeruginosa* ATCC 27853; and CAL, *Candida albicans* ATCC 14053.

Peak ROS intensity, measured as maximum RLU over the time course, varied by microbe and opsoni-zation status (Figure 4a). Serum opsonization significantly increased peak CL for *K. pneumoniae* 1706 (19.6 vs. 5.8 RLU, p < 0.05), 1705 (20.5 vs. 8.5 RLU), and 700603 (17.6 vs. 11.3 RLU). *S. aureus* and *C. albicans*, however, showed decr-eased peak CL after opsonization (by 5.4 and 3.3 RLU, respectively). *P. aeruginosa* showed the highest peak CL overall (36 RLU) with only a small difference between opsonized and non-opsonized forms. Except for *K. pneumoniae* 1706, peak ROS intensity did not significantly change with opsonization across other microbes.

#### Time to peak response

Time to peak ROS production further highlighted opsonization effects (Figure 4b). *K. pneumoniae* 1705 opsonized with serum peaked significantly faster (73 min) than non-opsonized samples (160 min) (p < 0.01). A similar acceleration was observed for *K. pneu-moniae* 1706 and 700603, as well as for *S. aureus* and *P. aeruginosa*. In contrast, *C. albicans* showed a delayed peak after opsonization (~13 min later). The only statistically significant change in peaking time was recorded for *K. pneumoniae* 1705.

## DISCUSSION

### Role of ROS and study objectives

The production of ROS by phagocytic leukocytes plays a critical role in the effective elimination of microbial pathogens [23]. The present study investigated microbial-specific ROS kinetics using whole blood from camels, offering a physiologically relevant approach to assess innate immune responses. Additionally, this study examined the effect of serum opsonization on ROS production, offering insights into the role of humoral factors in augmenting phagocyte responses.

### Comparative ROS kinetics among pathogens

In this study, we analyzed the *in vitro* kinetics of ROS production and peak time response in dromedary camel blood treated with opsonized or non-opsonized *K. pneumoniae*, *S. aureus*, *P. aeruginosa*, *C. albicans*, and *E. coli*. Notably, serum opsonization significantly enhanced ROS production and peak CL responses in *K. pneumoniae* strains compared to *S. aureus* and *C. albicans* strains treated similarly. On the other hand, the overall reduction in ROS, accompanied by a simultaneous increase in the peak CL response to *P. aeruginosa*, indicates a contrasting effect of serum opsonization on the ROS response to *P. aeruginosa*. Except for *C. albicans*, the opsonization of microbes with serum seemed to shorten the peaking time. The remarkable difference in the total amount of ROS produced among different microbes suggests a diversity in the microbes that stimulate ROS production in camel leukocytes, suggesting that Gram-negative bacteria may elicit a more potent ROS response than their Gram-positive counterparts. In line with this observation, a previous study by Johnson *et al*. [24] demonstrated that a higher percentage of neutrophils produced ROS against Gram-negative bacteria than Gram-positive bacteria.

### Influence of bacterial cell wall on ROS response

However, Gram-positive and Gram-negative bacteria are vulnerable to various antimicrobial mecha-nisms, as the cell wall structure dictates the initial tar-gets and the sequence of reactions leading to their killing. In line with this, recent investigations have indicated that *S. aureus* responds to the host environment by increasing the cell wall thickness. This was evident after incubating *S. aureus* with human serum, which led to a host-induced increase in cell wall thickness and a reduction in the exposure of bound antibody and complement, resulting in the downregulation of phagocytosis and neutrophil killing [25]. Considering the impact of the structural differences of bacteria, dielectric barrier discharge plasma treatments have demonstrated greater efficacy against Gram-negative bacteria (*Salmonella Typhimurium*) than against Gram-positive bacteria (*S. aureus*) [26]. In line with this, previous research by Richardson *et al*. [27] has shown that the bactericidal effect of the Fenton reaction required higher concentrations to kill Gram-positive bacteria (chemical generation of hydroxyl radical) than for Gram-negative bacteria (*P. aeruginosa*) as it may be that Gram-negative bacteria are more susceptible to hydroxyl radical.

### ROS production and virulence variability in *K. pneumoniae*

This study showed a noticeable difference in the total ROS production among the *K. pneumoniae* opsonized strains. A previous study by Simoons-Smit *et al* [28] reported an association between the pote-ntial of *K. pneumoniae* strains to induce ROS production and their virulence, with lower CL response in human leukocytes to higher virulent strains. In agreement with this, *K. pneumoniae* 700603, which produces extended-spectrum β-lactamases (ESBLs), making it resistant to β-lactams antibiotics [29], induced the lowest total ROS production among all strains tested in the present study. The stronger virulence and pathogenicity of ESBL-producing *K. pneumoniae* compared to non-ESBL-producing *K. pneumoniae*, as well as their higher potential for biofilm formation, have been reported in previous studies by Huang *et al*. [30] and Simoons-Smit *et al*. [28]. Conversely, *K. pneumoniae* 1706, which lacks the *bla_* *K. pneumoniae* carbapenemase (KPC) gene, induced lower total ROS production than *K. pneumoniae* 1705, a KPC producing strain. Similarly, *K. pneumoniae* 1705 elicited the highest total ROS response among all strains tested and is a KPC strain recognized for its broad-spectrum antibiotic resistance. KPC-producing strains exhibit resistance to both β-lactam and non-β-lactam classes of antibiotics. A previous study by Rasheed *et al*. [29] suggested that KPC production itself was not associated with increased virulence, as certain KPC-producing strains demonstrate serum resistance, whereas others remain serum-sensitive. In addition, certain immunological responses, such as phagocytic rates, in mice were not significantly correlated with KPC subtype; instead, they were associated with multilocus sequence typing and capsular serotype [31].

### Suppressed ROS production by *S. aureus*

The opsonized *S. aureus* strain exhibited relatively low total ROS production, as reflected by its CL kinetics. These results correlate with a study that states that killing Gram-negative bacteria is complement-mediated, whereas Gram-positive bacteria, such as *Streptococcus pneumoniae*, rely predominantly on opsonophagocytic uptake and complement-mediated immune responses [32]. Staphylococcal accessory ele-ment regulator/sensor system-regulated virulence factors in *S. aureus* suppress ROS generation in human neutrophils [33]. In addition, neutrophil itaconate (methyl succinate) production stimulated by *S. aureus* was found to impair the oxidative killing capacity of neutrophils against *S. aureus* by attenuating the respiratory burst [34], in light of which we speculate about our results of lower total ROS production against *S. aureus*. The low ROS response of camel leukocytes to *S. aureus* could be the result of their potential to interfere with the complement system, as previously reported by Rooijakkers *et al*. [35]. The study demonstrated that *S. aureus* complement inhibitors bind to C3 convertases, thereby interfering with the deposition of additional C3b on the bacteria through the classical, alternative, and lectin complement pathways, resulting in reduced phagocytosis and killing. Moreover, *S. aureus* Panton-Valentine Leukocidin targets the human complement receptors C5aR and C5L2, thereby inhibiting C5a-mediated immune cell activation and ultimately blocking neutrophil activation and detection of the microbe [36].

### ROS dynamics in opsonized *P. aeruginosa*

For serum – opsonized *P. aeruginosa* achieved peak ROS levels more rapidly than its non-opsonized counterpart, with a lower total ROS production than in the case without serum omission, indicating a more rapid immune activation following opsonization. This observation may be attributed to complement-facilitated phagocytosis of *P. aeruginosa* through the alternative pathway, as previously demonstrated by Mueller-Ortiz *et al*. [37] in mice that survived *P. aeru-ginosa*-induced acute pneumonia by activating the alternative pathway rather than the classical or lectin pathways. The reduced efficiency of phagocytosis of *P. aeruginosa* opsonized with serum from C3-defi-cient mice compared to serum from healthy mice also supports this [37]. Similarly, the observed decline of ROS response to opsonized *P. aeruginosa* at an earlier time interval of <90 min is consistent with a previous report by Mizgerd [38] indicating complete elimination of *P. aeruginosa* within 90 min by ROS produced from human WBCs in 90 min. In general, the observed high ROS response to both opsonized and non-opsonized *P. aeruginosa* confirms the esse-ntial role of ROS in the granzyme-mediated killing of *P. aeruginosa* by natural killer cells, as previously reported by Feehan *et al*. [39].

### Lack of ROS response to *E. coli* and methodological considerations

In contrast to a previous report by Hussen [14] demonstrating robust ROS induction by *E. coli* strains isolated from camel mastitis milk in camel granulocytes, the present study did not observe a significant ROS response to *E. coli*. This discrepancy may be attributed to strain-specific differences in the interaction between *E. coli* and the camel’s innate immune system. More-over, the difference may reflect the sensitivity and specificity of the ROS detection method used. While previous studies employed dihydrorhodamine 123 (DHR) assays, which primarily detect intracellular ROS, the present study used luminol-dependent CL, which is more sensitive to extracellular ROS production. Furth-ermore, a comparative study of neutrophil activation by zymosan using CL and DHR demonstrated differences in kinetic parameters between the two methods [40]. It is possible that *E. coli* triggered limited intracellular ROS, which would be detected by DHR, but failed to induce sufficient extracellular ROS detectable by CL. In addition, *E. coli* failed to sufficiently engage key neutrophil receptors or suppress downstream NADPH Nox2 activation. Furthermore, some *E. coli* strains are known to express antioxidant enzymes, such as super-oxide dismutase, which can neutralize ROS and further dampen the oxidative response [10]. Although *E. coli* did not elicit a CL response in the present study, preliminary data showed a 4-log reduction in *E. coli* growth after incubation with 100% camel blood (Unpublished data). The data suggest that alternative bactericidal mech-anisms, independent of ROS, may be responsible for *E. coli* elimination in the whole blood of camels.

### Limited ROS induction by *C. albicans*

In our study, total ROS production against *C. albicans* was found to be the lowest in both opso-nized and non-opsonized conditions compared to other microbes, consistent with a previous study by Moldovan *et al* [41], which reported limited oxidative burst activity against *C. albicans* in its yeast form. Moreover, the observed similar total ROS response to opsonized and non-opsonized *C. albicans* seems to cont-rast with the reported role of ROS production in the killing of opsonized *C. albicans* by human neutrophils, whereas neutrophil-mediated killing of non-opsonized *C. albicans* was ROS-independent [42].

### Implications for camel immunology and veterinary application

These findings highlight the differential oxidative burst responses to opsonized and non-opsonized micr-obes elicited by various pathogens in camel phagocytes, underscoring the variable effects of serum opsonization in inducing distinct immune responses against microbial infections. The results of the present study contribute to an improved understanding of the characteristics of the basal innate immune mechanisms in camels, including microbial-induced oxidative respiratory bursts, which are essential for understanding the mechanisms of disease pathogenesis. The usefulness of this study in veterinary practices lies in its potential for therapeutic interventions, which can be informed by understanding how different pathogens interact with the camel’s innate immune system.

## CONCLUSION

This study provides the first comprehensive evalu-ation of microbe-specific oxidative burst responses in dromedary camel (*C. dromedarius*) whole blood using a luminol-based CL assay. The results demonstrated that camel leukocytes exhibit distinct ROS production kinetics in response to different microbial pathogens, with serum opsonization modulating the magnitude and timing of the response. Among the tested pathogens, Gram-negative bacteria, particularly *K. pneumoniae* 1705 and *P. aeruginosa*, elicited the strongest ROS production, whereas Gram-positive *S. aureus* and fungal *C. albicans* triggered comparatively weaker responses. Notably, opsonization significantly enhanced ROS generation and accelerated peak response time in *K. pneumoniae* strains, while *P. aeruginosa* showed faster kinetics but reduced total ROS post-opsonization. *E. coli*, in contrast, failed to induce measurable extracellular ROS under the experimental conditions.

These findings have important implications for camel health management, particularly in the context of increasing AMR and zoonotic transmission risks. Understanding how camel phagocytes respond diffe-rently to various pathogens provides a foundation for improving diagnostic tools, vaccine design, and thera-peutic interventions, especially for high-risk bacterial infections such as *K. pneumoniae* and *P. aeruginosa*.

A major strength of this study lies in its use of physiologically relevant whole blood assays rather than isolated neutrophils, thereby preserving natural cellular interactions and avoiding artificial priming effects. Furthermore, the inclusion of clinically relevant microbial strains and the use of serum opsonization mimic *in vivo* immune conditions, enhancing translational relevance.

Despite these strengths, the study has certain limitations. The ROS detection was confined to extra-cellular measurement through luminol, potentially overlooking intracellular ROS dynamics. In addition, the study was limited to a single animal species and specific pathogen strains, which may not fully represent the variability observed in field infections. The mechanisms by which some pathogens, such as *E. coli*, evade ROS detection remain speculative and warrant further investigation.

Future studies should employ dual ROS detection methods (e.g., DHR for intracellular ROS), flow cyto-metry-based phagocytosis assays, and genetic profiling of immune response genes to gain a more complete picture of camel innate immunity. In addition, exploring ROS responses in different physiological or pathological states (e.g., mastitis and pneumonia) and comparing across camelid species could further elucidate spe-cies- and condition-specific immune dynamics.

This study advances our understanding of camel innate immunity by characterizing the microbial and opsonin-specific dynamics of ROS production in camel blood. The evidence underscores that camel phagocytes mount differential oxidative responses depending on pathogen type and opsonization status, reaffirming the complexity of host–pathogen interactions. These insights pave the way for tailored immunotherapeutic strategies and make a meaningful contribution to the broader field of comparative immunology and camel health research.

## AUTHORS’ CONTRIBUTIONS

SAA: Conceptualization, validation, design of methodology, project administration and funding, data analysis, and writing the original draft. NAK: Sample collection, conducting experiments, data collection and analysis, and writing the original draft. AAH: Sample collection, conducting experiments, reviewing and editing the original draft, and language proofreading. SAB and SMAH: Sample collection, reviewing and editing the original draft, and language proofreading. JH, WAM, and YET: Reviewing and editing the original draft and language proofreading. All authors have read and approved the final manuscript.
